# Genome-Enabled Prediction Models for Yield Related Traits in Chickpea

**DOI:** 10.3389/fpls.2016.01666

**Published:** 2016-11-22

**Authors:** Manish Roorkiwal, Abhishek Rathore, Roma R. Das, Muneendra K. Singh, Ankit Jain, Samineni Srinivasan, Pooran M. Gaur, Bharadwaj Chellapilla, Shailesh Tripathi, Yongle Li, John M. Hickey, Aaron Lorenz, Tim Sutton, Jose Crossa, Jean-Luc Jannink, Rajeev K. Varshney

**Affiliations:** ^1^Research Program-Grain Legumes, International Crops Research Institute for the Semi-Arid TropicsHyderabad, India; ^2^Division of Genetics, Indian Agricultural Research InstituteDelhi, India; ^3^Australian Centre for Plant Functional Genomics, University of AdelaideAdelaide, SA, Australia; ^4^The Roslin Institute and Royal (Dick) School of Veterinary Studies, The University of EdinburghEaster Bush, UK; ^5^Department of Agronomy and Horticulture, University of NebraskaLincoln, OR, USA; ^6^Crop Improvement, South Australian Research and Development InstituteUrrbrae, SA, Australia; ^7^International Maize and Wheat Improvement CenterMexico, Mexico; ^8^School of Integrative Plant Science, Cornell UniversityIthaca, NY, USA; ^9^School of Plant Biology and Institute of Agriculture, The University of Western AustraliaWestern Australia WA, Australia

**Keywords:** genomic prediction accuracy, genetic gain, genomic selection, chickpea, training population, population structure, prediction models

## Abstract

Genomic selection (GS) unlike marker-assisted backcrossing (MABC) predicts breeding values of lines using genome-wide marker profiling and allows selection of lines prior to field-phenotyping, thereby shortening the breeding cycle. A collection of 320 elite breeding lines was selected and phenotyped extensively for yield and yield related traits at two different locations (Delhi and Patancheru, India) during the crop seasons 2011–12 and 2012–13 under rainfed and irrigated conditions. In parallel, these lines were also genotyped using DArTseq platform to generate genotyping data for 3000 polymorphic markers. Phenotyping and genotyping data were used with six statistical GS models to estimate the prediction accuracies. GS models were tested for four yield related traits *viz*. seed yield, 100 seed weight, days to 50% flowering and days to maturity. Prediction accuracy for the models tested varied from 0.138 (seed yield) to 0.912 (100 seed weight), whereas performance of models did not show any significant difference for estimating prediction accuracy within traits. Kinship matrix calculated using genotyping data reaffirmed existence of two different groups within selected lines. There was not much effect of population structure on prediction accuracy. In brief, present study establishes the necessary resources for deployment of GS in chickpea breeding.

## Introduction

Chickpea (*Cicer arietinum*) is the second largest cultivated grain legume globally which plays vital role in ensuring food and nutritional security in Asian and sub-Saharan African regions of the world. Because of its higher protein content chickpea serves as an important source of protein in vegetarian diet. Chickpea also fits well in crop rotation programs because of its ability to fix atmospheric nitrogen and improve the soil nutritional profile. It is a self-pollinated, diploid (*2n* = 16) annual crop with genome size of ~740 Mbp (Varshney et al., 2013a). Currently the chickpea is grown over 14.80 Mha area across the 55 countries globally, accounting for an annual production of 14.24 million tons (FAOSTAT, 2014). Average chickpea productivity is <1 t ha^−1^ which is much lower than its potential yield of 6 t ha^−1^ under optimum growing conditions, due to its exposure to several biotic and abiotic stresses including *Ascochyta* blight, *Fusarium* wilt, drought, heat, and salinity. Among these stresses, terminal drought is one of the major yield constraints that solely is responsible for about 40% yield loss (Ahmad et al., 2005). Conventional breeding approaches coupled with genomics-assisted breeding have been successful to some extent in enhancing the productivity from 0.60 t ha^−1^ in 1960s to 0.96 t ha^−1^ in 2014 (FAOSTAT, 2014). However, this is not enough to meet the demand for exponentially growing world population. Therefore, there is a need to deploy genomics-assisted breeding approaches e.g., genomic selection (GS) for chickpea improvement (Varshney et al., 2005).

Recent advances in the next generation sequencing (NGS) and high-throughput genotyping technologies provide an opportunity for translating genomics information in crop breeding (Varshney et al., 2014a, 2015). Until few years back chickpea was considered as one of the orphan crops with respect to genomic resources. At present, large scale genome resources including simple sequence repeats (SSRs; Thudi et al., 2011), single nucleotide polymorphisms (SNPs; Hiremath et al., 2012), genetic maps, and genotyping platforms are available in chickpea (Varshney et al., 2012a). In order to exploit the potential of SNP markers in the molecular breeding applications, highly informative SNP markers were converted in to cost-effective VeraCode assays to be used in chickpea genetics and breeding applications (Roorkiwal et al., 2013). In addition, very recently draft genome sequences have also become available for kabuli (Varshney et al., 2013a) and desi (Jain et al., 2013; Ruperao et al., 2014) type. In addition, very recently draft genome for wild chickpea (*C. reticulatum*) has also become available (Gupta et al., 2016).

In terms of translational genomics for agriculture, improved breeding lines have been developed for drought tolerance (Varshney et al., 2013b) and disease resistance (Varshney et al., 2013c) by using marker-assisted backcrossing (MABC) approach. For addressing complex traits such as yield under rainfed conditions that are generally governed by many small effect QTLs, MABC is not a very effective approach (Ribaut and Ragot, 2007), whereas GS approach using genome-wide marker profile has been suggested as a potential breeding approach for developing superior lines to address such complex traits (Meuwissen et al., 2001; Varshney et al., 2012b).

In the GS approach, testing (prediction) population are not phenotyped but genotyped, and therefore breeding cycle time is reduced and genetic gain per unit time is enhanced. In plants, Bernardo and Yu (2007) were the first to show the utility of GS in terms of genetic gains as compared to marker-assisted selection using simulated data. Since the seminal work of Meuwissen et al. (2001) on GS, a number of studies on assessing the prediction accuracy in different economically important crops, using different marker platforms and marker densities [including genotyping by sequencing, (GBS)] applying different parametric and non-parametric statistical models, have been published so far (de los Campos et al., 2009, 2010; Crossa et al., 2010, 2011; Jannink et al., 2010; González-Camacho et al., 2012; Dawson et al., 2013). Both, simulation and empirical studies have shown that GS has higher prediction accuracy than standard pedigree-based prediction, and most of the benefits of GS arise from obtaining accurate predictions at early stages of the breeding cycle (i.e., rapid cycling of selection). Choice of model, the size of the training population, the heritability of the trait, the span of linkage disequilibrium (LD), the marker density, and the strength of the genetic relationships between the training and validation populations, are some other majors factors known to affect genomic predictions.

Majority of GS studies in crops had emphasis on estimation of prediction accuracy using simulated data and suggested up to 40% better efficiency than marker assisted selection (MAS; Bernardo and Yu, 2007). In the case of winter wheat, efficiencies of selecting line using phenotypic selection (PS), conventional MAS, and GS across 13 different agronomic traits were compared and average prediction accuracy for GS was found 28% higher as compared to MAS, as accurate as PS for selecting the lines (Heffner et al., 2011). Similarly, in the case of pea, Illumina GoldenGate SNPs chip has been used for assessing prediction values in the pea diversity panel comprising of 367 accessions (Burstin et al., 2015). In rice, GS analysis was performed on 363 elite breeding lines using five-fold GS cross-validation (Spindel et al., 2015). GS has been effectively used in the hybrid breeding programs of wheat (Rutkoski et al., 2012; Zhao et al., 2014), maize (Windhausen et al., 2012), and rice (Xu et al., 2014). Empirical selection experiment using a maize bi-parental with temperate and tropical maize indicated the clear advantages of GS in terms of genetic gains per unit of time (years) over marker assisted recurrent selection (MARS) and/or conventional selection. For grain yield and stover quality traits, GS resulted in 14–50% gains than MARS (Massman et al., 2013). Beyene et al. (2015) compared GS with pedigree selection across eight biparental tropical maize populations evaluated in drought stressed environments, and reported that the average gain per cycle from GS across the eight bi-parental populations was 0.086 Mg ha^−1^. Semagn et al. (2015) reported that the average gain per cycle using MARS across 10 populations was 0.045 Mg ha^−1^ under drought stressed conditions. Based on the potential of GS for developing superior lines with higher yield under rainfed conditions and enhancing the genetic gains ultimately in chickpea breeding, the present study was planned to identify the suitable GS models for predicting breeding values using genome wide markers on elite breeding lines in chickpea.

## Materials and methods

### Phenotyping

A set of 320 elite breeding lines from the International Chickpea Screening Nursery (ICSN), which was tested for yield and yield related traits at several locations for many years were used in this study (Table S1). These lines included both desi and kabuli seed types. The whole set was phenotyped extensively for several yield and yield related traits at two locations in India namely, IARI, New Delhi, and ICRISAT, Patancheru during crop seasons 2011–12 and 2012–13. An incomplete block design was planted with three replications per environment under irrigated and rainfed condition. Due to seed limitation IARI during year I (2011–12) undertook only one rainfed experiment. However, during year II (2012–13), because of heavy rain at the time of sowing both the seasons were treated as irrigated.

Following four traits were selected for detailed analysis for each year at both the locations.

- Days to flowering (DF): Number of days to achieve 50% flowering in whole plot.
- Days to maturity (DM): Number of days to achieve the maturity in whole plot.
- 100 seed weight (SDW): Plants were harvested and random 100 seeds were weighed.
- Seed yield (SY): Plants from each plot were harvested and weighed to measure the seed yield.

All these four traits were further used for random cross validation prediction using GS models.

### DNA isolation and marker genotyping

Plant leaves were collected from 15 days old seedlings and genomic DNA was isolated using high throughput mini-DNA extraction method as described by Cuc et al. (2008). DNA was assessed using spectrophotometer (Shimadzu UV160A, Japan) for quantification and quality. On the basis on the quality, 315 lines (162 entries of desi and 153 entries of kabuli type) were selected for genotyping.

All selected lines were genotyped using sequencing-based DArT genotyping platform known as DArTseq as described in Sansaloni et al. (2011). In brief, complexity reduction methods optimized at DArT P/L were used. Site specific barcoded adapters were used for sequencing the DNA samples on a single lane of Illumina Genome Analyzer IIx (Illumina Inc., San Diego, CA). FASTQ files resulting from the sequencing run were filtered, split into their respective target (individual) data using barcode splitting script and aligned. Using an analytical pipeline developed by DArT P/L, alignment data was processed to produce “DArT score” tables and “SNP” tables (Sansaloni et al., 2011). The DArTseq method deploys sequencing of the representations on the NGS platforms and generated two types of data (i) SilicoDArTs calculated as dominant (presence/absence) markers, and (ii) SNPs in fragments present in the representation (http://www.diversityarrays.com/dart-application-dartseq-data-types).

### Data analysis

Phenotyping data on 320 lines generated at IARI and ICRISAT was curated and used for further analysis. Analysis of Variance (ANOVA) for phenotypic data was performed for the targeted traits (DM, DF, SDW, and SY) using SAS software version 9.4 (SAS Institute, 2013). Best Linear Unbiased Predictors (BLUP) of entries were estimated considering replication as fixed and nested block effect and entry factor as random using model

(1) $$\begin{array}{l} y_{ijk}=\mu +r_{i}+{\left(r/b\right)}_{ij}+g_{k}+\varepsilon_{ijk} \end{array}$$

Where *y*_*ijk*_ is the phenotypic trait analyzed; μ is the grand mean; *r*_*i*_ is the fixed effect of replication *i*; (*r*/*b*)_*ij*_ is the random effect of block *j* nested with replication *i* with N $\left(0,\;I\sigma_{b}^{2}\right)$; *g*_*k*_ is the random effect of entry *k* with N $\left(0,\;I\sigma_{g}^{2}\right)$ and ε_*ijk*_ is the random residual effect with N $\left(0,\;I\sigma_{\varepsilon }^{2}\right)$. Broad-sense heritability was calculated for each trait using method for unbalanced trials (Piepho and Möhring, 2007). The coefficient of variations at phenotypic and genotypic level variation was calculated following Johnson et al. (1955). Combined ANOVA for each location was performed across different years using residual maximum likelihood (REML) procedure by making the error variances homogeneous. The model used to calculate the adjusted means across environments was

(2) $$\begin{array}{l} y_{ijkl}=\mu +e_{i}+{\left(e/r\right)}_{ij}+{\left(e/r/b\right)}_{ijk}+g_{l}+{\left(eg\right)}_{il}+\varepsilon_{ijkl} \end{array}$$

Where *y*_*ijkl*_ is the phenotypic trait analyzed; μ is the grand mean; *e*_*i*_ is the fixed effect of year *i*; (*e*/*r*)_*ij*_ is the random effect of replication *j* in year *i* with N $\left(0,\;I\sigma_{r}^{2}\right)$; (*e*/*r*/*b*)_*ijk*_ is the random effect of block *k* nested with replication *j* in year *i* with N $\left(0,\;I\sigma_{b}^{2}\right)$; *g*_*l*_ is the random effect of entry *l* with N $\left(0,\;I\sigma_{g}^{2}\right)$; (*eg*)_*il*_ is the random effect of the interaction between entry *l* year *i* with N $\left(0,\;I\sigma_{eg}^{2}\right)$ and ε_*ijkl*_ is the random residual effect with N $\left(0,\;I\sigma_{\varepsilon }^{2}\right)$.

Genotyping data for 3000 polymorphic markers including SilicoDArTs and DArT-SNP markers were analyzed collectively for 315 elite lines. Marker statistics such as polymorphism information content (PIC) value, gene diversity, minor allele frequency (MAF) and missing percentage were calculated using PowerMarker V3.0 (Liu and Muse, 2005). DARwin-5.0 program (Perrier et al., 2003) was used to construct a tree using unweighted neighbor joining method to examine the genetic structure and diversity existing in population undertaken. Linkage disequilibrium was measured by the parameter *r*^2^ calculated as

(3) $$\begin{array}{l} r^{2}=\frac{{\left(P_{AB}P_{ab}-P_{Ab}P_{aB}\right)}^{2}}{P_{A}P_{B}P_{a}P_{b}} \end{array}$$

where pA, pB, pa, and pb are the frequencies of alleles A, B, a, and b in the population. Haplotype frequencies of allele combinations are denoted as pAB, pAb, paB, and pab, respectively. *r*^2^ and LD decay were computed using an R package Synbreed.

### Prediction models

Six different models including Ridge Regression Best Linear Unbiased Predictor (RR-BLUP), Kinship GAUSS (semiparametric model), Bayes Cπ, Bayes B, Bayesian Least Absolute Shrinkage, and Selection Operator (Bayesian LASSO) and Random Forest (RF) (machine learning algorithm) were used for prediction of GEBVs. RR-BLUP assumes that all markers have common variances with small but non-zero effect and therefore shrinks equally for each marker effect (Meuwissen et al., 2001). Bayesian based methods such as Bayes Cπ assumes a common marker effect variance for all markers which follows a scaled inverse prior with parameters (Habier et al., 2011). Bayes B method assumes that only a proportion of the markers explain total genetic variance and most other markers explain zero variance (Meuwissen et al., 2001). Bayes B method considers every marker for estimating the variance using a prior distribution that assumes that this variance is small and has a predefined probability. Bayesian LASSO method estimates a marker specific shrinkage based on a regularization parameter. The RF algorithm is a collection of classifications on bootstrap subsets aiming to capture non-additive effects (Heslot et al., 2012). RF was implemented using the R package “RandomForest” (Liaw and Wiener, 2002). All analyses were performed in R 3.0.2 (R Core Team, 2013).

### Effect of missing marker data and MAF on prediction accuracy

In order to assess the impact of marker attributes *viz* missing marker data and MAF on prediction accuracies, genotyping data for 315 elite lines along with the phenotyping data for 100 seed weight for ICRISAT location was taken under consideration. Nine different combinations of missing marker data and MAF (including markers in combination with 0%, ≤10%, and ≤30% missing data, and 0%, ≥5%, and ≥10% MAF) were used with all six different GS models for estimating prediction accuracies. For calculating the prediction accuracy, marker effects were calculated using standard linear model using following equation:

(4) $$\begin{array}{l} y=F\psi +X\beta +e \end{array}$$

where *y* denotes the vector of adjusted phenotypes of order and ψ is a px1 vector of fixed effects, *F* is a known incidence matrix corresponding to fixed effects, *X* is a matrix of genotypes for markers, β is the vector of marker effects and *e* is a vector of random residual terms.

### Estimation of model prediction accuracy

For fitting the GS model, separate analyses were performed for four yield and yield related traits *viz*. DM, DF, SDW, and SY for both locations and seasons as well. To estimate the prediction accuracy of GEBV, the approach of cross-validation (CV) was employed. Five-fold cross-validation was performed to predict the breeding values in different environments and also in pooled environment conditions. Five-fold CV was performed by randomly assigning 80% of the lines as training population and the remaining 20% as testing candidates. The whole process was repeated 20 times, resulting in a total of 100 CV runs. The prediction accuracy was measured as Pearson correlation between the observed adjusted phenotypic values (i.e., BLUP) and the prediction values computed by the different models.

### Effect of population structure on prediction accuracy

In order to assess the effect of population structure and population size on GEBV/prediction accuracy, population structure was considered as one of the factor for calculating the prediction accuracy. With genotyping data on 315 elite lines, number of natural genetic groups (K) and the distribution of individuals among these groups were estimated using STRUCTURE 2.3 (Pritchard et al., 2000). Based on the number of groups identified using diversity and STRUCTURE analysis, GEBVs were calculated for each group. Prediction accuracy were estimated individually for each group and population structure, K matrix were included during the prediction accuracy analysis.

## Results

### Descriptive interpretation of phenotyping data

Phenotyping data for two different treatments *viz*. irrigated (IR) and rainfed (RF) at ICRISAT and IARI for five seasons were used to calculate coefficient of variation (CV), genetic variance (GV), phenotypic coefficient of variation (PCV), genotypic coefficient of variation (GCV), and broad sense heritability (*H*^2^) and environmental coefficient of variation (ECV) for yield and yield related traits *viz*. Days to flowering (DF); Days to maturity (DM); 100 seed weight (SDW; g); Seed yield (SY; g per plot; Table 1). Significant differences were observed in DF and DM pattern of both the locations (ICRISAT and IARI) with higher values in IARI. High broad sense heritability up to 0.99 was observed for all the four traits (Table 1). The highest variability (GCV and PCV) was recorded for SY and the lowest for DM (Table 1).

**Table 1:** **Analysis of variance (ANOVA) and genetic estimates for days to flower, days to maturity, 100 seed weight and seed yield**.

**Trait**	**Seasons**	**Mean**	***SD***	**CV**	**GV**	***H^2^***	**GCV**	**PCV**	**ECV**
Days to flowering (DF)	ICRISAT-IR-12	38.93	2.13	5.48	30.20	0.95	14.12	15.14	5.48
	ICRISAT-IR-13	42.60	2.29	5.39	30.62	0.94	12.99	14.06	5.39
	ICRISAT-RF-13	44.76	2.10	4.69	8.29	0.84	6.43	7.96	4.69
	IARI-IR-12	66.46	0.81	1.22	232.01	0.99	22.92	22.95	1.22
	IARI-IR-13	65.48	0.33	0.50	606.18	0.99	37.60	37.61	0.50
Days to maturity (DM)	ICRISAT-IR-12	103.11	2.05	1.99	1.73	0.54	1.28	2.36	1.99
	ICRISAT-IR-13	93.93	1.86	1.98	14.63	0.92	4.07	4.53	1.98
	ICRISAT-RF-13	91.62	2.60	2.84	8.98	0.79	3.27	4.33	2.84
	IARI-IR-12	153.24	0.93	0.61	11.74	0.96	2.24	2.32	0.61
	IARI-IR-13	153.16	0.18	0.12	12.29	0.99	2.29	2.29	0.12
100 seed weight (SDW; g)	ICRISAT-IR-12	26.65	1.17	4.39	57.16	0.99	28.36	28.70	4.39
	ICRISAT-IR-13	28.44	1.69	5.95	67.32	0.99	28.84	29.45	5.95
	ICRISAT-RF-13	28.96	2.29	7.92	68.81	0.98	28.64	29.72	7.92
	IARI-IR-12	32.29	0.52	1.62	68.59	0.99	25.65	25.70	1.62
	IARI-IR-13	27.12	0.35	1.31	67.99	0.99	30.40	30.43	1.31
Seed yield (SY; g per plot)	ICRISAT-IR-12	122.13	13.55	11.10	1318.21	0.95	29.73	31.73	11.10
	ICRISAT-IR-13	134.47	18.94	14.09	385.92	0.76	14.61	20.29	14.09
	ICRISAT-RF-13	119.23	14.20	11.91	567.20	0.89	19.97	23.26	11.91
	IARI-IR-12	140.50	35.42	25.21	2854.12	0.82	38.03	45.62	25.21
	IARI-IR-13	233.98	5.20	2.22	10304.00	0.99	43.38	43.44	2.22

*SD, Standard deviation; CV, Coefficient of variation; GV, Genetic Variance; GCV, Genotypic Coefficient of Variation; H^2^, Heritability (broad sense); PCV, Phenotypic Coefficient of Variation; ECV, Environmental Coefficient of variation*.

### Polymorphism features and linkage disequilibrium across the population

**Figure 1:**
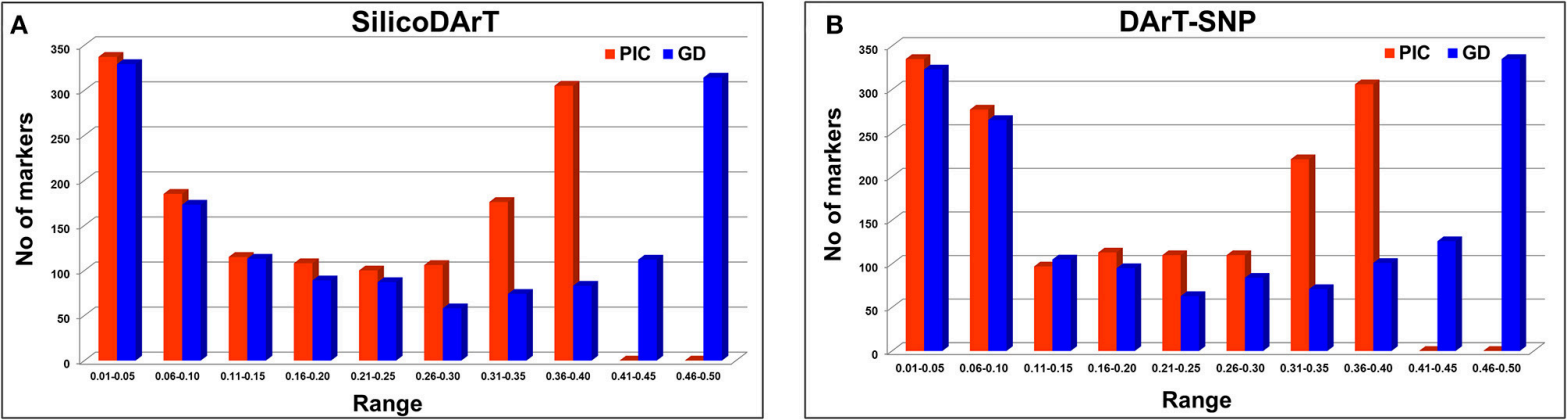
**Estimation of polymorphism information content (PIC) value and gene diversity of markers used (A)** SilicoDArTs **(B)** DArT-SNP markers.

As mentioned in the Methods section, 315 chickpea lines were selected on the basis of genomic DNA quality from a set of 320 elite breeding lines. These lines were genotyped using sequencing-based DArT genotyping platform known as DArTSeq. In total 1432 SilicoDArTs and 1568 DArT-SNP markers were found polymorphic across the lines. As expected these lines are elite breeding lines with very low genetic diversity, estimated PIC value ranged from 0.01 to 0.38 for SilicoDArTs across these genotypes with a mean PIC value of 0.20 (Figure 1A; Table S2). Gene diversity of these SilicoDArTs across these lines ranged from 0.01 to 0.50 with a mean gene diversity value of 0.24 (Figure 1A; Table S2). However, in the case of DArT-SNPs, the PIC value ranged from 0.01 to 0.38 across the genotypes with a mean PIC value of 0.19 (Figure 1B; Table S3).

**Figure 2:**
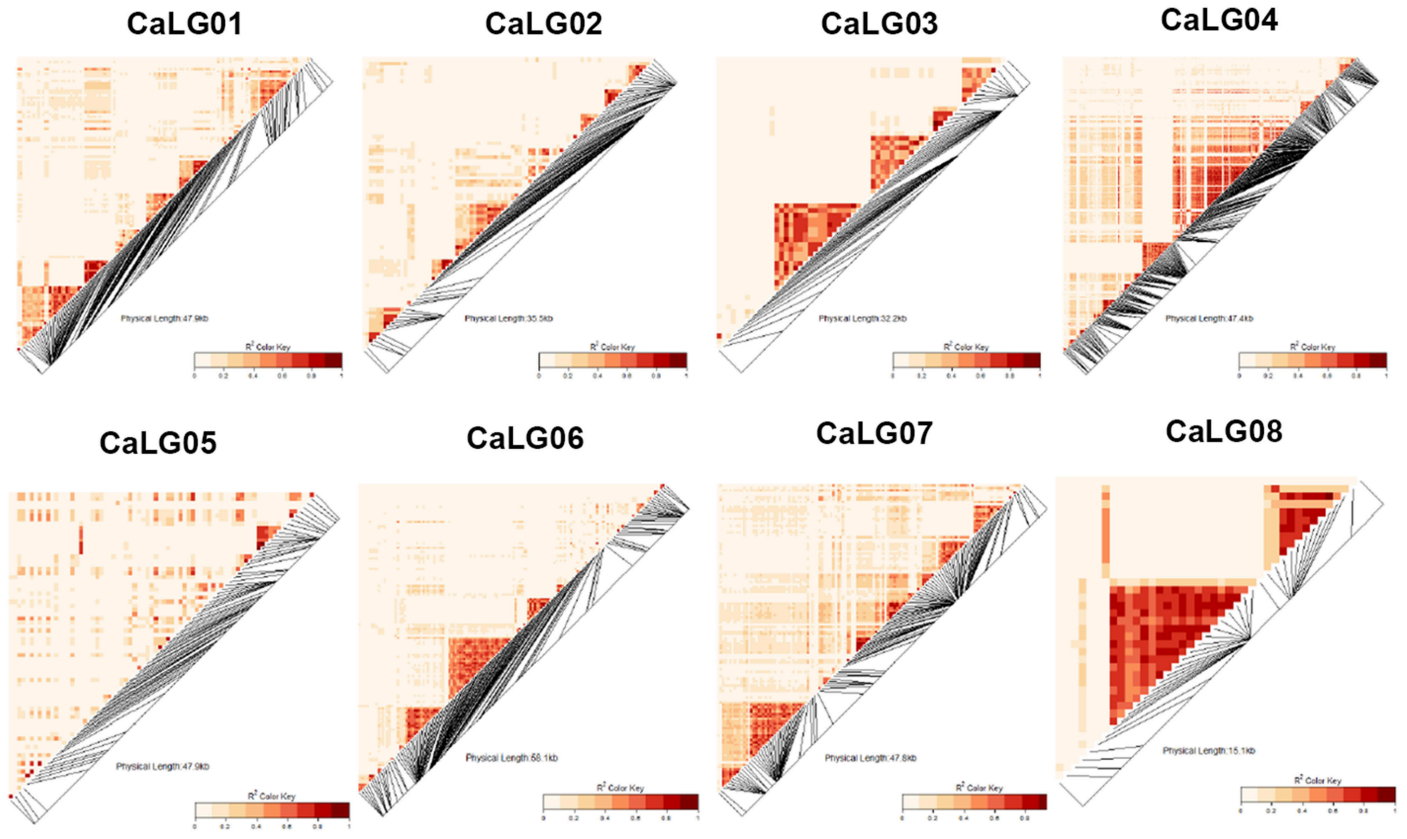
**Genome-wide LD heat map constructed using SilicoDArTs and DArT-SNP markers across the 315 elite lines of chickpea**. Linkage disequilibrium was calculated using *r*^2^ = 0.2 as threshold. Genome-wide LD extend up to 500–2500 kb in CaLG02 and CaLG04. One huge LD block on CaLG04 was observed.

**Figure 3:**
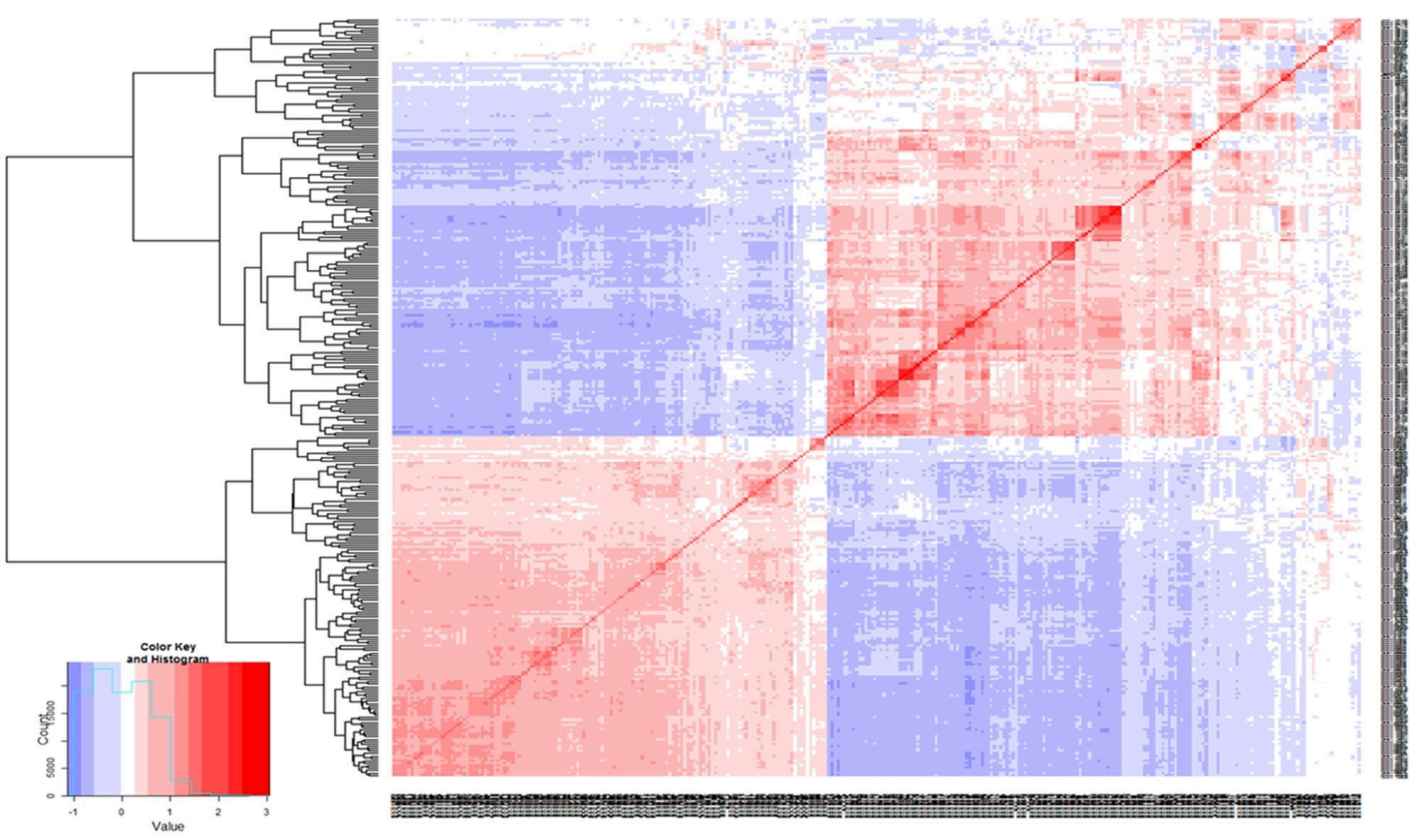
**Genetic relatedness among the 315 elite lines**. Distance matrix was calculated using average linkage clustering. Heat map of the kinship matrix and dendrogram analysis using genotyping data clearly depicts the existence of two different group among the elite lines.

Marker sequences for SilicoDArTs and DArT-SNPs were aligned to the kabuli reference genome (Varshney et al., 2013a) for identifying the physical map position of markers and selected set of 970 markers [combined set of SilicoDArTs (633) and DArT-SNPs (337)] were used for LD analysis. Using *r*^2^ = 0.2 as threshold, LD was found extending up to 500–2500 kb on CaLG02 and CaLG04 (Figure 2; Figure S1). One huge LD block was observed on CaLG04 (Figure 2). The heat map developed using kinship matrix showed a very close relationship within these lines and reaffirms existence of two different groups existing among these 315 lines that is possibly attributed by two different seed types of chickpea; i.e., desi and kabuli (Figure 3).

### Effect of missing marker data and minor allele frequency on prediction accuracy

Different prediction accuracies were obtained with 9 different combination of percentage of missing marker data and minor allele frequency (MAF; Table 2). The best prediction accuracy (0.713) was obtained for Random Forest model with combination of markers including all markers with 0% missing marker data and ≥5% MAF, whereas lowest accuracy (0.655) was obtained for Bayes B model on including markers with 0% missing marker data and ≥10% MAF (Table 2). However, for further analysis combination of genotyping data with ≤30% missing marker data and marker data with ≥10% MAF was used based on consistency of results on repetitive analysis.

**Table 2:** **Effect of missing marker data and minor allele frequency on prediction accuracy**.

**Method**	**0% missing**	**0% missing**	**0% missing**	**≤10% missing**	**≤10% missing**	**≤10% missing**	**≤30% missing**	**≤30% missing**	**≤30% missing**
	**- 0% MAF**	**- ≥5% MAF**	**- ≥10% MAF**	**- 0% MAF**	**- ≥5% MAF**	**- ≥10% MAF**	**- 0% MAF**	**- ≥5% MAF**	**- ≥10% MAF**
Ridge Regression	0.681	0.679	0.669	0.669	0.676	0.671	0.660	0.674	0.670
Kinship Gauss	0.697	0.710	0.702	0.692	0.701	0.695	0.688	0.698	0.702
Bayes Cπ	-	0.678	0.662	-	0.688	0.674	-	0.668	0.683
Bayes B	-	0.674	0.655	-	0.680	0.660	-	0.663	0.663
Bayes LASSO	0.660	0.681	0.666	0.684	0.671	0.665	0.672	0.657	0.680
Random Forest	0.694	0.713	0.705	0.709	0.694	0.693	0.698	0.689	0.697

*Miss, Missing marker data; MAF, Minor Allele Frequency*.

### Cross comparison of performance of different GS model

Six different statistical methods used in the present study for each of the four traits, were selected on the basis of their demonstrated ability to estimate the GEBVs. Performance of models was found to vary across the different seasons and traits, however there were not much difference in performance of models within a season for an individual trait (Table 3). In general, high prediction accuracies were observed for DF and SDW, however prediction accuracies for DM and SY were varying for different locations and seasons (Table 3; Figure S2). For DF and SDW, highest prediction accuracies were obtained for ICRISAT-IR-12 and least for ICRISAT-RF-13. In case of DM and SY highest prediction accuracies were obtained for ICRISAT-IR-13 and IARI-IR-12, respectively (Table 3).

**Table 3:** **Comparative analysis of prediction accuracies of different GS models for four yield related traits across chickpea population**.

		**ICRISAT-IR-13**		**ICRISAT-RF-13**		**IARI-IR-13**		**IARI-IR-12**		**ICRISAT-IR-12**	
	**Methods**	**Correlations**	**SE**	**Correlations**	**SE**	**Correlations**	**SE**	**Correlations**	**SE**	**Correlations**	**SE**
Days to flowering (DF)	Ridge Regression	0.665	0.005	0.556	0.006	0.674	0.005	0.663	0.006	0.823	0.003
	Kinship Gauss	0.707	0.005	0.635	0.005	0.673	0.005	0.701	0.006	0.847	0.003
	Bayes Cπ	0.663	0.005	0.564	0.006	0.675	0.005	0.663	0.006	0.824	0.003
	Bayes B	0.647	0.005	0.560	0.006	0.673	0.005	0.664	0.006	0.825	0.003
	Bayes LASSO	0.666	0.005	0.562	0.006	0.673	0.005	0.664	0.006	0.827	0.003
	Random Forest	0.693	0.005	0.626	0.006	0.683	0.004	0.695	0.006	0.851	0.003
Days to maturity (DM)	Ridge Regression	0.794	0.004	0.478	0.006	0.301	0.008	0.325	0.009	0.374	0.007
	Kinship Gauss	0.808	0.004	0.539	0.006	0.304	0.008	0.320	0.008	0.394	0.007
	Bayes Cπ	0.799	0.004	0.495	0.006	0.304	0.009	0.324	0.009	0.379	0.007
	Bayes B	0.798	0.004	0.510	0.006	0.289	0.009	0.331	0.009	0.395	0.007
	Bayes LASSO	0.797	0.004	0.476	0.006	0.301	0.008	0.329	0.009	0.376	0.007
	Random Forest	0.815	0.004	0.531	0.007	0.254	0.009	0.300	0.009	0.407	0.007
100 seed weight (SDW)	Ridge Regression	0.893	0.002	0.797	0.004	0.816	0.004	0.898	0.002	0.909	0.002
	Kinship Gauss	0.893	0.002	0.798	0.003	0.817	0.004	0.909	0.002	0.912	0.002
	Bayes Cπ	0.892	0.002	0.797	0.003	0.817	0.004	0.901	0.002	0.909	0.002
	Bayes B	0.887	0.002	0.792	0.004	0.816	0.004	0.903	0.002	0.908	0.002
	Bayes LASSO	0.892	0.002	0.799	0.004	0.817	0.004	0.900	0.002	0.909	0.002
	Random Forest	0.897	0.002	0.801	0.004	0.815	0.004	0.909	0.001	0.912	0.002
Seed yield (SY)	Ridge Regression	0.523	0.006	0.172	0.008	0.166	0.008	0.604	0.005	0.222	0.008
	Kinship Gauss	0.522	0.006	0.148	0.008	0.138	0.008	0.602	0.005	0.218	0.008
	Bayes Cπ	0.520	0.007	0.175	0.008	0.163	0.008	0.602	0.005	0.216	0.008
	Bayes B	0.517	0.006	0.171	0.008	0.168	0.008	0.597	0.005	0.209	0.009
	Bayes LASSO	0.524	0.006	0.182	0.008	0.163	0.007	0.598	0.006	0.216	0.008
	Random Forest	0.493	0.006	0.186	0.008	0.165	0.009	0.606	0.005	0.205	0.009

*SE, Standard Error*.

There was no overall best performer or underperformer model while estimating prediction accuracies. For instance, in DF trait, Kinship Gauss model was found best performer for three seasons (ICRISAT-IR-13: 0.707; ICRISAT-RF-13: 0.635; IARI-IR-12: 0.701) and for the rest two seasons Random Forest model was found producing highest prediction accuracies (IARI-IR-13: 0.683; ICRISAT-IR-12: 0.851). While the least prediction accuracy was observed with RR BLUP [ICRISAT-RF-13: 0.556; IARI-IR-12: 0.663 (equivalent to Bayes Cp); ICRISAT-IR-12: 0.823], Bayes B (ICRISAT-IR-13: 0.647); IARI-IR-13: 0.673 (equivalent to Bayes LASSO; Table 3).

### Impact of population structure on estimation of GEBVs

Prediction accuracies were estimated for all 315 elite lines altogether considering them as a single set as well as separately as two groups by considering desi and kabuli type. Further, to estimate the effect of population structure on the prediction accuracy, prediction accuracies were also estimated by taking the population structure in account. It was observed that prediction accuracies calculated individually for desi and kabuli seed type varied to a large extent when compared with the prediction accuracy for all 315 lines. For instance, in the case of DF, maximum prediction accuracy observed was 0.851, whereas when calculated individually using groups of desi and kabuli lines showed significantly lower prediction accuracy values (0.681 and 0.573, respectively; Table 4). Similarly for DM and SY, variations in prediction accuracies were observed when it was calculated separately for desi and kabuli type.

**Table 4:** **Effect of population structure/size on prediction accuracy using six GS models for yield related traits**.

**Methods**	**ICRISAT-IR-13**			**ICRISAT-RF-13**			**IARI-IR-13**			**ICRISAT-RF-12**			**IARI-IR-12**		
	**All**	**kabuli**	**desi**	**All**	**kabuli**	**desi**	**All**	**kabuli**	**desi**	**All**	**kabuli**	**desi**	**All**	**kabuli**	**desi**
**Days to flowering (DF)**															
Ridge Regression	0.665 ± 0.005	0.531 ± 0.011	0.639 ± 0.007	0.556 ± 0.006	0.418 ± 0.012	0.646 ± 0.007	0.674 ± 0.005	0.350 ± 0.017	0.377 ± 0.011	0.823 ± 0.003	0.477 ± 0.014	0.561 ± 0.010	0.663 ± 0.006	0.451 ± 0.011	0.518 ± 0.009
Kinship Gauss	0.707 ± 0.005	0.572 ± 0.010	0.637 ± 0.007	0.635 ± 0.005	0.444 ± 0.011	0.673 ± 0.007	0.673 ± 0.005	0.374 ± 0.018	0.366 ± 0.011	0.847 ± 0.003	0.510 ± 0.014	0.572 ± 0.009	0.701 ± 0.006	0.474 ± 0.011	0.507 ± 0.009
Bayes Cπ	0.663 ± 0.005	0.532 ± 0.011	0.635 ± 0.007	0.564 ± 0.006	0.425 ± 0.011	0.652 ± 0.007	0.675 ± 0.005	0.285 ± 0.018	0.373 ± 0.011	0.824 ± 0.003	0.413 ± 0.015	0.567 ± 0.010	0.663 ± 0.006	0.420 ± 0.011	0.507 ± 0.009
Bayes B	0.647 ± 0.005	0.527 ± 0.012	0.605 ± 0.007	0.560 ± 0.006	0.404 ± 0.012	0.629 ± 0.008	0.673 ± 0.005	0.283 ± 0.018	0.375 ± 0.010	0.825 ± 0.003	0.328 ± 0.016	0.567 ± 0.010	0.664 ± 0.006	0.449 ± 0.012	0.475 ± 0.009
Bayes LASSO	0.666 ± 0.005	0.539 ± 0.011	0.638 ± 0.007	0.562 ± 0.006	0.413 ± 0.011	0.645 ± 0.007	0.673 ± 0.005	0.341 ± 0.018	0.378 ± 0.011	0.827 ± 0.003	0.469 ± 0.013	0.566 ± 0.009	0.664 ± 0.006	0.432 ± 0.012	0.509 ± 0.009
Random Forest	0.693 ± 0.005	0.573 ± 0.011	0.624 ± 0.007	0.626 ± 0.006	0.441 ± 0.011	0.681 ± 0.007	0.684 ± 0.005	0.373 ± 0.017	0.363 ± 0.010	0.851 ± 0.003	0.443 ± 0.016	0.614 ± 0.008	0.695 ± 0.006	0.436 ± 0.013	0.516 ± 0.009
**Days to maturity (DM)**															
Ridge Regression	0.794 ± 0.004	0.499 ± 0.011	0.392 ± 0.013	0.478 ± 0.007	0.490 ± 0.011	0.385 ± 0.010	0.301 ± 0.008	0.096 ± 0.014	0.378 ± 0.011	0.374 ± 0.007	0.356 ± 0.014	0.254 ± 0.011	0.325 ± 0.009	0.195 ± 0.014	0.371 ± 0.010
Kinship Gauss	0.808 ± 0.004	0.508 ± 0.010	0.390 ± 0.013	0.539 ± 0.006	0.480 ± 0.010	0.405 ± 0.011	0.304 ± 0.008	0.099 ± 0.014	0.370 ± 0.011	0.394 ± 0.007	0.354 ± 0.015	0.293 ± 0.010	0.320 ± 0.008	0.195 ± 0.015	0.378 ± 0.011
Bayes Cπ	0.799 ± 0.004	0.489 ± 0.011	0.368 ± 0.013	0.496 ± 0.006	0.474 ± 0.011	0.375 ± 0.011	0.304 ± 0.009	0.053 ± 0.014	0.362 ± 0.011	0.379 ± 0.007	0.350 ± 0.014	0.248 ± 0.011	0.324 ± 0.009	0.188 ± 0.013	0.369 ± 0.010
Bayes B	0.798 ± 0.004	0.456 ± 0.012	0.369 ± 0.013	0.510 ± 0.006	0.453 ± 0.012	0.346 ± 0.011	0.289 ± 0.009	0.072 ± 0.014	0.352 ± 0.011	0.395 ± 0.007	0.355 ± 0.016	0.239 ± 0.010	0.331 ± 0.009	0.176 ± 0.015	0.356 ± 0.012
Bayes LASSO	0.797 ± 0.004	0.505 ± 0.011	0.386 ± 0.013	0.476 ± 0.006	0.476 ± 0.012	0.384 ± 0.010	0.301 ± 0.008	0.049 ± 0.013	0.367 ± 0.011	0.376 ± 0.007	0.354 ± 0.015	0.259 ± 0.010	0.329 ± 0.009	0.150 ± 0.014	0.377 ± 0.011
Random Forest	0.815 ± 0.004	0.466 ± 0.012	0.375 ± 0.011	0.531 ± 0.007	0.448 ± 0.012	0.405 ± 0.010	0.254 ± 0.009	0.056 ± 0.015	0.346 ± 0.011	0.407 ± 0.007	0.341 ± 0.016	0.288 ± 0.010	0.300 ± 0.009	0.179 ± 0.014	0.354 ± 0.010
**100 seed weight (SDW)**															
Ridge Regression	0.893 ± 0.002	0.609 ± 0.009	0.678 ± 0.008	0.797 ± 0.004	0.548 ± 0.009	0.512 ± 0.010	0.816 ± 0.004	0.441 ± 0.013	0.335 ± 0.013	0.909 ± 0.002	0.701 ± 0.008	0.726 ± 0.007	0.898 ± 0.002	0.641 ± 0.008	0.732 ± 0.007
Kinship Gauss	0.893 ± 0.002	0.626 ± 0.008	0.676 ± 0.008	0.798 ± 0.004	0.530 ± 0.009	0.506 ± 0.010	0.817 ± 0.004	0.443 ± 0.013	0.325 ± 0.013	0.912 ± 0.002	0.718 ± 0.007	0.723 ± 0.008	0.909 ± 0.002	0.672 ± 0.008	0.731 ± 0.006
Bayes Cπ	0.892 ± 0.002	0.611 ± 0.009	0.656 ± 0.008	0.797 ± 0.003	0.551 ± 0.010	0.509 ± 0.011	0.817 ± 0.004	0.442 ± 0.012	0.343 ± 0.012	0.909 ± 0.002	0.708 ± 0.007	0.715 ± 0.008	0.901 ± 0.002	0.637 ± 0.009	0.726 ± 0.007
Bayes B	0.887 ± 0.002	0.588 ± 0.009	0.630 ± 0.009	0.792 ± 0.004	0.559 ± 0.009	0.501 ± 0.010	0.816 ± 0.004	0.445 ± 0.012	0.375 ± 0.012	0.908 ± 0.002	0.688 ± 0.007	0.699 ± 0.009	0.903 ± 0.002	0.646 ± 0.008	0.704 ± 0.007
Bayes LASSO	0.892 ± 0.002	0.614 ± 0.008	0.674 ± 0.008	0.799 ± 0.004	0.553 ± 0.009	0.514 ± 0.010	0.817 ± 0.004	0.442 ± 0.013	0.332 ± 0.012	0.909 ± 0.002	0.703 ± 0.008	0.727 ± 0.008	0.900 ± 0.002	0.632 ± 0.010	0.735 ± 0.007
Random Forest	0.897 ± 0.002	0.647 ± 0.007	0.725 ± 0.007	0.801 ± 0.004	0.562 ± 0.009	0.556 ± 0.010	0.815 ± 0.004	0.478 ± 0.013	0.319 ± 0.014	0.912 ± 0.002	0.727 ± 0.008	0.745 ± 0.008	0.909 ± 0.001	0.652 ± 0.009	0.742 ± 0.007
**Seed yield (SY)**															
Ridge Regression	0.523 ± 0.006	0.267 ± 0.012	0.261 ± 0.012	0.172 ± 0.008	0.093 ± 0.013	0.063 ± 0.011	0.166 ± 0.008	0.153 ± 0.013	0.243 ± 0.011	0.222 ± 0.008	0.053 ± 0.013	0.241 ± 0.011	0.604 ± 0.005	0.399 ± 0.010	0.697 ± 0.006
Kinship Gauss	0.522 ± 0.006	0.218 ± 0.012	0.251 ± 0.013	0.148 ± 0.008	0.062 ± 0.011	0.177 ± 0.012	0.138 ± 0.008	0.199 ± 0.012	0.232 ± 0.012	0.218 ± 0.008	0.023 ± 0.013	0.242 ± 0.012	0.603 ± 0.005	0.453 ± 0.009	0.690 ± 0.006
Bayes Cπ	0.520 ± 0.007	0.246 ± 0.013	0.262 ± 0.012	0.175 ± 0.008	−0.004 ± 0.011	−0.002 ± 0.011	0.163 ± 0.008	0.144 ± 0.012	0.236 ± 0.012	0.216 ± 0.008	−0.060 ± 0.012	0.235 ± 0.011	0.602 ± 0.005	0.403 ± 0.010	0.687 ± 0.006
Bayes B	0.517 ± 0.006	0.227 ± 0.013	0.285 ± 0.012	0.171 ± 0.008	−0.003 ± 0.012	−0.010 ± 0.011	0.168 ± 0.008	0.141 ± 0.013	0.227 ± 0.011	0.209 ± 0.009	−0.063 ± 0.012	0.239 ± 0.011	0.597 ± 0.005	0.406 ± 0.012	0.675 ± 0.006
Bayes LASSO	0.524 ± 0.006	0.262 ± 0.013	0.262 ± 0.012	0.182 ± 0.008	0.004 ± 0.012	0.001 ± 0.010	0.163 ± 0.007	0.143 ± 0.012	0.240 ± 0.011	0.216 ± 0.008	−0.061 ± 0.012	0.247 ± 0.012	0.598 ± 0.006	0.408 ± 0.010	0.690 ± 0.006
Random Forest	0.493 ± 0.006	0.190 ± 0.012	0.258 ± 0.012	0.186 ± 0.008	0.104 ± 0.012	0.077 ± 0.011	0.165 ± 0.009	0.192 ± 0.013	0.131 ± 0.011	0.205 ± 0.009	0.161 ± 0.012	0.186 ± 0.013	0.606 ± 0.005	0.457 ± 0.010	0.655 ± 0.007

However, while estimating the prediction accuracy for SDW, the prediction accuracies were consistently lower when calculated for desi and kabuli seed type in comparison to single set of 315 lines. For instance, the highest prediction accuracy obtained for SDW was 0.912 when calculated using 315 elite lines as single group, while prediction accuracies when calculated individually for desi and kabuli groups, were found reducing at a lesser extent; i.e., 0.742 and 0.727, respectively, in comparison to other traits (Table 4).

**Figure 4:**
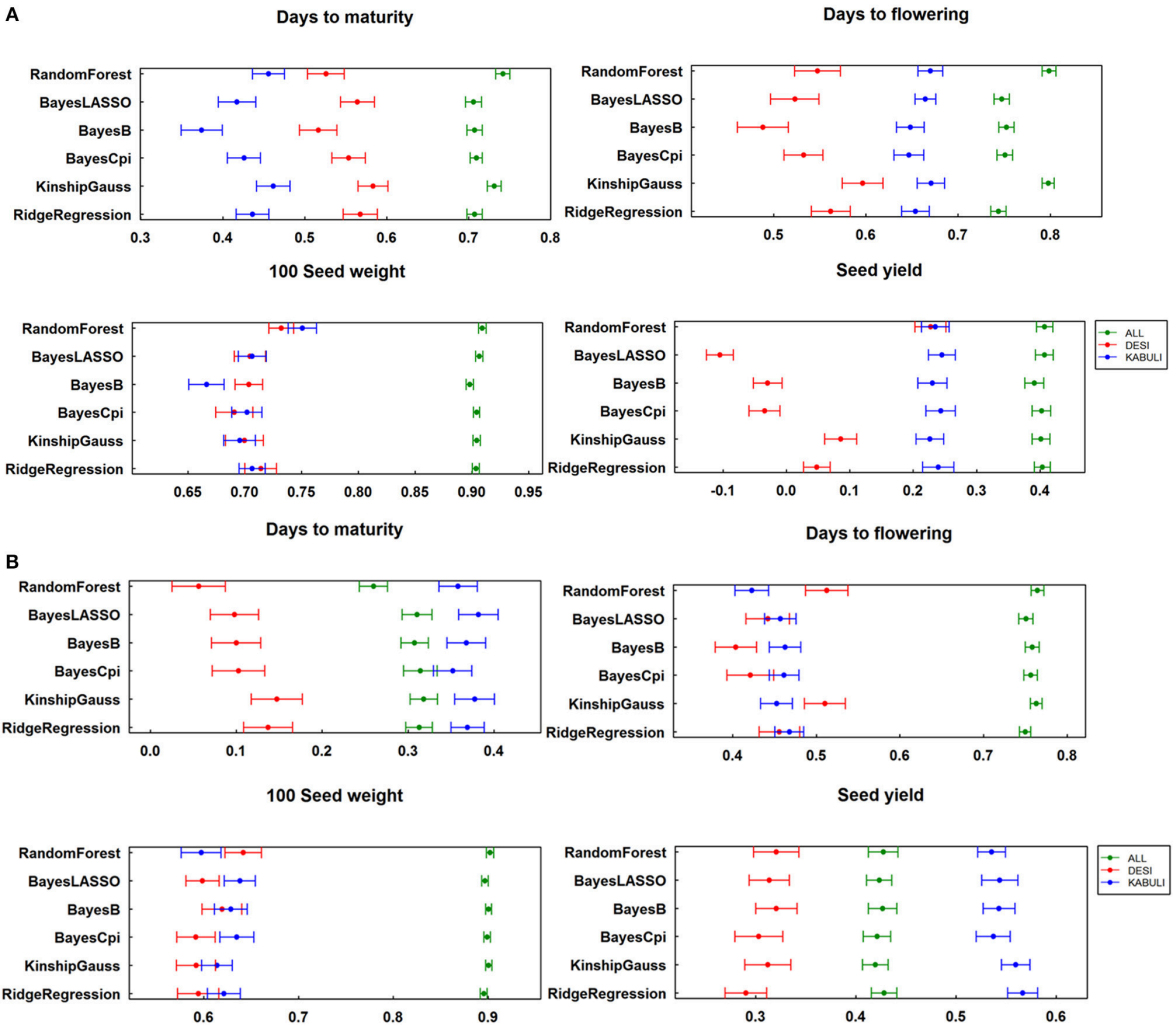
**Regression of true breeding value on breeding values estimated with different methods (A)** for ICRISAT location; **(B)** for IARI location.

Similarly to assess the effect of population structure on the prediction accuracy, calculated “K” matrix was included in the script as one of the variable while calculating the GEBVs. The prediction accuracies estimated by considering the population structure showed slight increase in value (Figure S2). In addition to prediction accuracy, another measuring factor, regression coefficients were calculated for all traits across both locations by considering 315 elite lines together as one group and desi and kabuli groups separately. For data from three seasons at ICRISAT, regression coefficients for DM, DF, and SY varied significantly when compared at whole population level and at individual group level, like prediction accuracies. However, the regression coefficients were found comparatively stable for SDW (Figure 4A). In a similar manner, two season's data at IARI showed variable regression coefficients for DM, DF, and SY when dealing with desi, kabuli groups and all lines separately (Figure 4B).

## Discussion

Integration of genomics tools in conventional breeding is offering efficient, cost, and time effective, precise solution for agriculture to fulfill current and future food demands as well as crisis arising due to changing global environment. In order to enhance the chickpea productivity, efforts are being done with conventional and modern breeding approaches. At molecular level various advances have been made using available resources. For instance using linkage mapping a genomic region “*QTL-hotspot*” was identified on CaLG04 of chickpea that harbor several QTLs for controlling the drought tolerance related root traits and several other yield related traits (Varshney et al., 2014b). Following studies have indicated the role of several small effect QTLs for conferring drought tolerance in chickpea (Jaganathan et al., 2015; Kale et al., 2015). Successful identification and mapping of several drought responsive gene(s)/genomic region(s) (Roorkiwal et al., 2014; Thudi et al., 2014) further widen the scope of selection of genomic regions for breeding purposes. Efforts to introgress the identified “*QTL-hotspot*” in to elite chickpea cultivar JG 11 using MABC approach have resulted in development of improved introgression lines with higher root traits [rooting depth (RDp), root length density (RLD), and root dry weight (RDW)] as compared to the recurrent (JG 11) as well as donor parent (ICC 4958) (Varshney et al., 2013b). Application of the first generation molecular breeding approaches such as MABC has resulted in enhanced yield under drought but still there is huge gap between actual and potential yield for chickpea. As discussed earlier success of MABC is limited to the simple traits but not to the complex traits (Ribaut and Ragot, 2007). Thus, efforts to use modern breeding approaches such as genomic selection with the ability to contribute to simple as well as complex traits are already underway to enhance the rate of genetic gain for various crops and livestock (Goddard et al., 2010; Heffner et al., 2010; Gorjanc et al., 2015).

With the availability of draft genome sequences (Jain et al., 2013; Varshney et al., 2013a; Ruperao et al., 2014; Gupta et al., 2016) and re-sequencing data for several hundred lines in chickpea, millions of markers have become available now. Ability of GS to address the complex traits and availability of increasing genomic resources enabling the application of emerging markers system like GBS and SNP array for estimating the prediction accuracy, sets the rationale for deployment of this molecular breeding tool for chickpea improvement. Selection of complex traits for the study was completely dependent on the nature of traits i.e., SDW under rainfed and irrigated condition provides a direct measure of drought tolerance and other three traits (DM, DF, SDW, and SY) are important yield parameters (Varshney et al., 2014b).

GS is known to enhance genetic gain with model using marker information as compared to pedigree based models. G × E interaction across different environments is also believe to significantly affect the genetic variability which controls the marker effect estimates (Crossa et al., 2010). Factors that affect prediction accuracy for estimating the GEBVs include statistical models (Heslot et al., 2012), number and type of molecular markers (Chen and Sullivan, 2003; Poland and Rife, 2012), linkage disequilibrium (Habier et al., 2007), effective population size (Daetwyler et al., 2008), relationship between calibration and test set (Pszczola et al., 2012) and population structure (Windhausen et al., 2012). In general, populations with higher genetic diversity require larger size of population for achieving better precision in GEBVs (Mujibi et al., 2011). Several studies have suggested to have minimum of 100–150 training population size for obtaining the optimum prediction accuracy (Bernardo and Yu, 2007). Considering these factors, we undertook a population with size of >300 lines that included two different seed types; i.e., desi and kabuli, each having >150 lines for optimal estimation of prediction accuracy (Table S1). High prediction accuracies were observed in the current study hence revalidating the results obtained in other crop plants in chickpea.

Small training populations of self-crossing reproduction species and/or bi-parental cross derived populations results in high prediction accuracies for GS analysis (Nakaya and Isobe, 2012), whereas application of further larger population size undertaken in the current study could also be attributed as an important factor for obtaining higher prediction accuracies. Inclusion of only elite lines in the current study could also be one of the critical factor in obtaining the higher prediction accuracies, hence selection of appropriate germplasm can also be considered as important factor as having a direct impact on prediction accuracies for GS.

Another important factor affecting the prediction accuracy is extent of LD decay which defines the minimum number of markers required for estimation of prediction accuracy. In the case of non-inbred lines there is a significant decrease in LD therefore requires large number of markers to compensate the fast decay in LD (Liu et al., 2015). LD calculated using squared-allele frequency correlations (*r*^2^; when *r*^2^ < 0.20) with mapped markers extended upto 500 kb (CaLG02)–2500 kb (CaLG04). Training population used in the present study included the elite breeding lines, LD analysis using genotyping data for these lines suggested presence of the huge LD blocks which could be one of the reasons for such high prediction accuracy (Table 3).

In general higher prediction accuracies are observed for the traits with less complexity while accuracy decreases with increase in the trait complexity (Zhang et al., 2014). In the current study, best prediction accuracy was observed for SDW (Table 3; Figure S2) as the trait is known to be less affected by other factors such as G × E interaction and treatments. Possible reason of SY having lower prediction accuracy in comparison to DM, DF, and SDW could be the variable nature of trait seed yield that is affected by several factors including G × E (Kashiwagi et al., 2006), which further affect prediction accuracy. Another possible reason for lower GS accuracy for SY could be that genomic region affecting the trait might not have been covered in the current genotyping data. Six different models (RR-BLUP, Kinship Gauss, Bayes Cπ, Bayes B, Bayes LASSO, and Random Forest) used for four yield and yield related traits including DM, DF, SDW, and SY using genotyping data from 970 SilicoDArTs and DArT-SNPs. Large variations observed in prediction accuracies were due to comparison made across the seasons and locations. However, there were not much variation in prediction accuracies across different models when comparing with-in same season and/or location. To some extent, Bayesian based methods and ridge regression models were found slightly stable as compared to rest others. Our results are supports earlier reports that suggests more or less similar performance of different models (Jannink et al., 2010). Few other studies comparing cross validation for different GS models suggested that trait genetic architecture did not affect similarity in performance of the model and most of the linear models like ridge regression and hierarchical Bayesian methods perform similarly (Heslot et al., 2012). Based on simulation data, Iwata and Jannink (2011) suggested the superiority of ridge regression methods over the Bayesian methods.

Further higher prediction accuracies could be the design of the study in such a manner that training set as well as testing set were phenotyped in same environment (Burstin et al., 2015). Diversity and population structure analysis using mapped markers suggested the presence of two different groups whereas no significant impact of population structure on prediction accuracy was observed. Our results were in complete accordance with results obtained in GS study on pea (Burstin et al., 2015).

Cattle breeding is one of the major beneficiaries of GS revolution and similar approach is being implemented in plant breeding for enhancing the rate of genetic gain by reducing the long duration selection cycles and increasing the selection intensity and efficiency. GS can play significant role in improving the traits with longer generation cycle and complex mechanism involving large number of small effect QTLs. Preliminary work in genomic selection for chickpea improvement has produced encouraging results with application of DArT markers system. It further opens a possibility to deploy high density genotyping methods like GBS and SNP arrays, which may result in more improvement in prediction and finally enhancement in the rate of genetic gain in chickpea.

## Author contributions

MR generated the genotyping data; MS, SS, PG, BC, and ST generated phenotyping data; MR, AR, and RD conducted phenotyping, genotyping data analysis including the statistical modeling; MR, AR, AJ, YL, JH, AL, TS, JC, JJ, and RV contributed to analyze and interpret data; MR, JC, and RV wrote the manuscript; RV conceived, designed, and supervised the study and finalized the manuscript.

### Conflict of interest statement

The authors declare that the research was conducted in the absence of any commercial or financial relationships that could be construed as a potential conflict of interest.
