# Supplementary figures and images for: Genome-Enabled Prediction Models for Yield Related Traits in Chickpea
Source: Front Plant Sci. 2016 Nov 22;7:1666. doi: 10.3389/fpls.2016.01666 (PMC5118446; doi:10.3389/fpls.2016.01666)

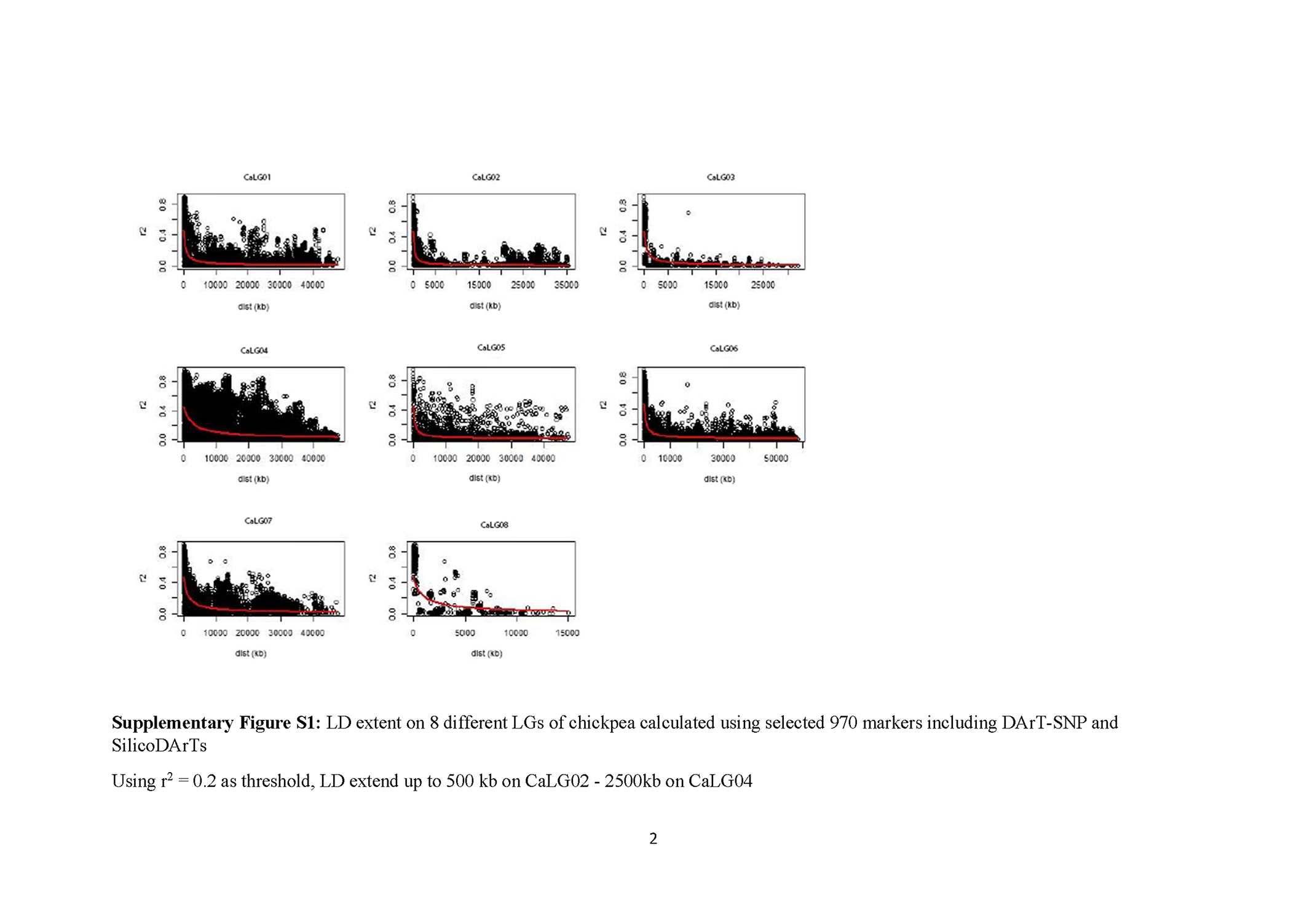

Supplement: Supplementary file 4 [file Image1.tiff]

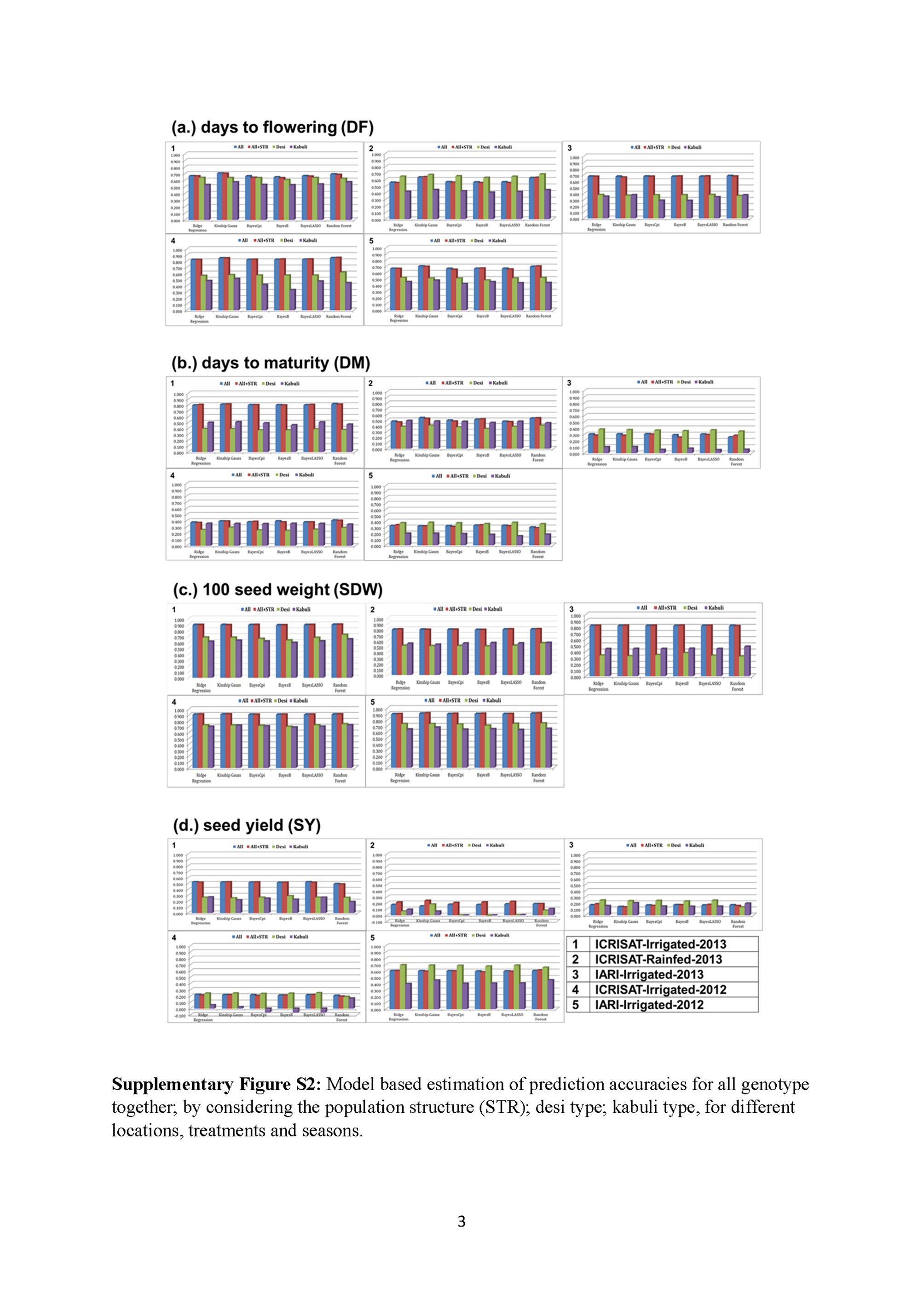

Supplement: Supplementary file 5 [file Image2.tiff]
